# Supplementary material for: A2AR eGFP reporter mouse enables elucidation of A2AR expression dynamics during anti-tumor immune responses
Source: Nat Commun. 2023 Nov 1;14:6990. doi: 10.1038/s41467-023-42734-0 (PMC10620403; doi:10.1038/s41467-023-42734-0)
Supplement: Supplementary file 1 — Supplementary Information [file 41467_2023_42734_MOESM1_ESM.pdf]

Supplementary Figure 1

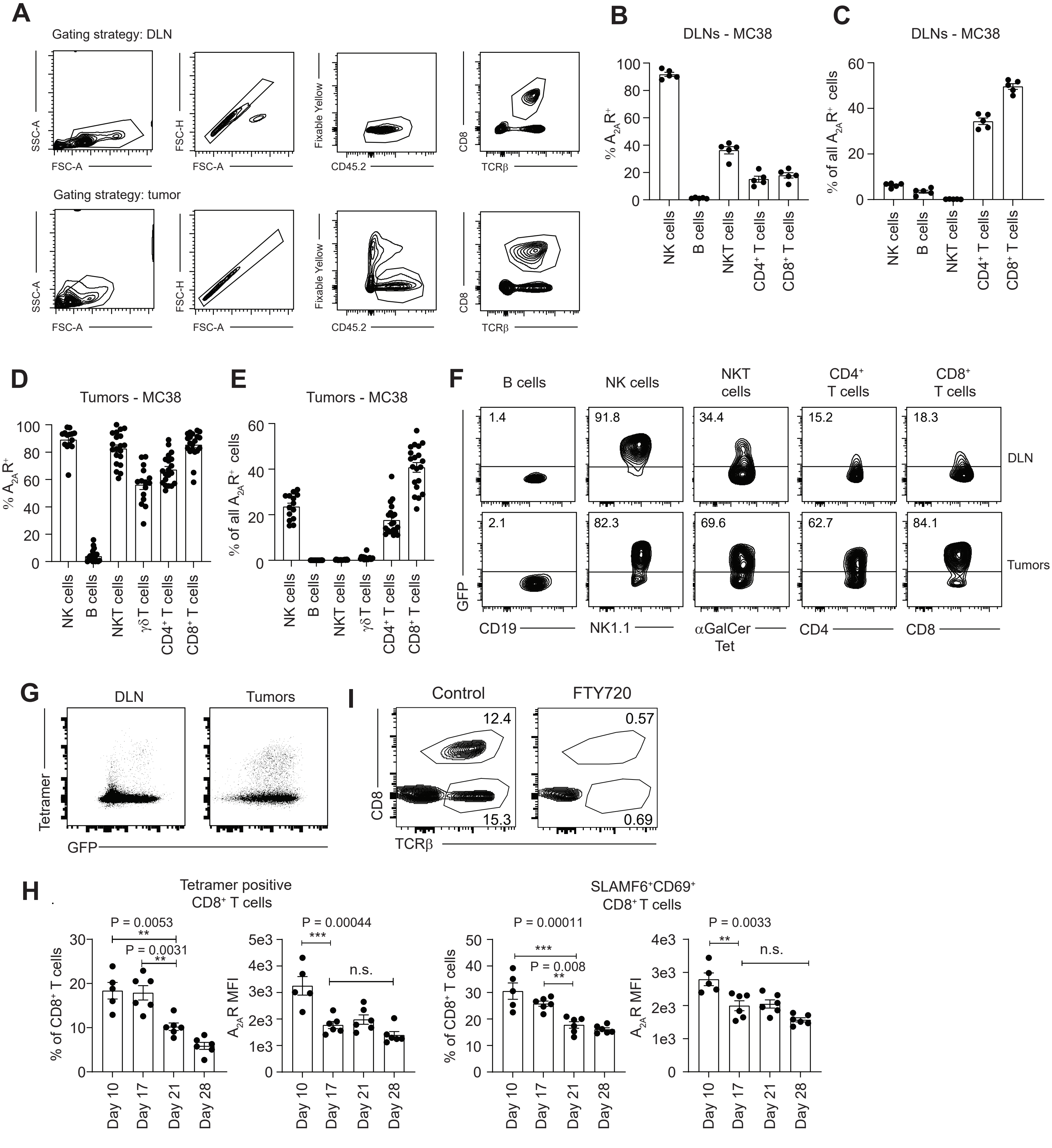

Supplementary Figure 1. Expression of A_{2A}R in tumor-infiltrating and draining lymph node resident lymphocytes.

C57BL/6 A_{2A}R GFP mice were injected with 5 x 10^5 AT3 ova tumor cells (**A, G-I**) or 1 x 10^6 MC38 tumor cells (**B-F**). At day 14 post tumor inoculation expression of GFP (A_{2A}R) was determined on tumor-infiltrating or draining lymph node resident lymphocytes. **A.** Gating strategy for $CD8^+$ T cells in the draining lymph nodes and tumors. **B,D.** Percentage of indicated subsets expressing GFP in the draining lymph nodes (**B**) or tumors (**D**). (**B-C**) (n=5), (**D-E**) NK cells (n= 14), γδ T cells (n= 15), B cells, $CD4^+$ T cells, and $CD8^+$ T cells (n= 20). **C**. Proportion of all GFP^+ cells that are of indicated lineage within draining lymph nodes (**C**) or tumors (**E**). **B-E**. Data represents the mean ± SEM of n = 5- 20 mice per group from up to 3 individual experiments. **F-G**. Representative flow cytometry plots of concatenated samples from a representative experiment showing GFP (A_{2A}R) expression in indicated subsets or expression profile vs SIINFEKL tetramer staining. **H**. Analysis of $CD8^+$ T cells from timecourse experiment in **Figure 2I**. Day 10 (n = 5), day 17, 21 and 28 (n = 6). **p<0.01, ***p<0.001, one way ANOVA. **I**. Mice were treated with PBS or FTY720 as per **Figure 3**. Analysis of the proportion of $TCR\beta^+CD8^+$ and $TCR\beta^+CD8^-$ cells in the blood of treated mice. Data represents a concatenated sample from a representative experiment. Source data are provided as a Source Data file.

Supplementary Figure 2

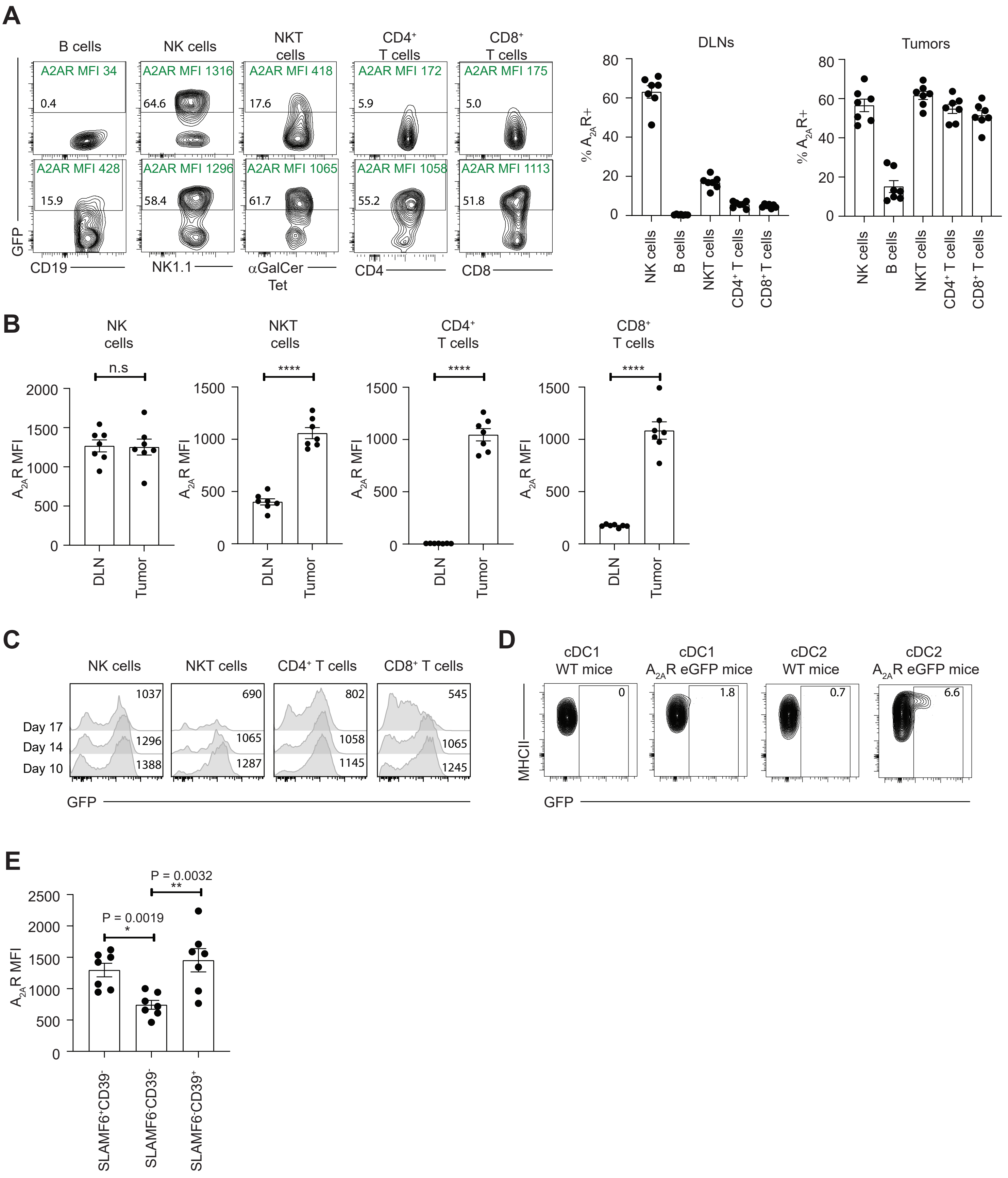

Supplementary Figure 2. Expression of $A_{2A}R$ in the E0771 tumor model

C57BL/6 $A_{2A}R$ GFP mice or wild-type mice were injected with 5 x 10^5 E0771 tumors sub-cutaneously. **A**. Expression of GFP on indicated subsets at day 14 post tumor injection. Left- representative flow cytometry plots based upon concatenated samples. Right- Mean ± SEM of n = 7 mice per group. **B**. MFI of $A_{2A}R$ (GFP) expression in indicated samples. Data represented as the mean ± SEM of n = 7 mice per group. **C**. Expression of $A_{2A}R$ (GFP) in indicated subsets over time. Values indicate GFP MFI. **D**. Expression of $A_{2A}R$ (GFP) in cDC1 and cDC2 cells isolated from tumors at day 14 post tumor inoculation. **E**. Expression of $A_{2A}R$ (GFP) in indicated subsets at day 14 post tumor inoculation. Data represented as the mean ± SEM of n = 7 mice per group. **A, C, D**. Representative staining from concatenated samples. *p<0.05 **p<0.01, ****p<0.0001, one way ANOVA (**E**) or paired student's t test (**B**). Source data are provided as a Source Data file.

Supplementary Figure 3

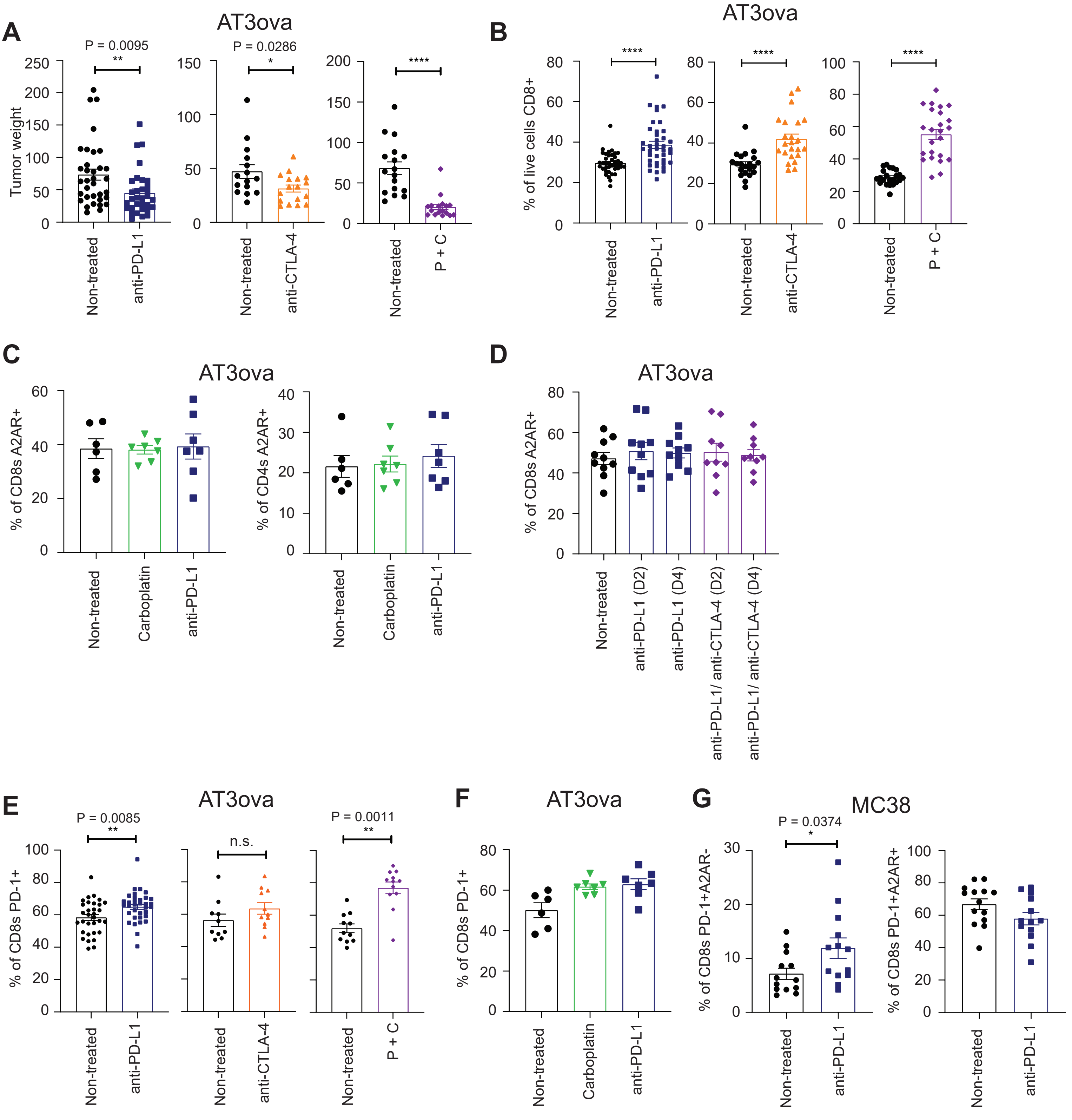

Supplementary Figure 3. Response of AT3 ova and MC38 tumors to immune checkpoint blockade.

C57BL/6 $A_{2A}R$ GFP mice were injected with 5 x 10^5 AT3 ova tumor cells (**A-F**) or 1 x 10^6 MC38 tumor cells (**G**). Where indicated mice were treated with anti-PD-L1, anti-CTLA-4 or both anti-PD-L1 and anti-CTLA-4 (P + C) as per **Figure 4.** Alternatively, where indicated mice were treated with 10 mg/ kg Carboplatin at day 14 post tumor inoculation. **A-C, E-G.** Tumor-infiltrating lymphocytes were analzyed at day 7 post treatment. **A-C**. Data represented as the mean ± SEM. **A**. Non-treated n = 34, anti-PD-L1 n = 32, anti-CTLA-4 n = 17, P + C n = 17 pooled from 7 individual experiments **B**. Non-treated n = 39, anti-PD-L1 n = 39, anti-CTLA-4 n = 23, P + C n = 24 pooled from 7 individual experiments. **C**. Data represented as the mean ± SEM of n = 6 (non-treated) or 7 (carboplatin or anti-PD-L1) mice per group. **D**. Timecourse analysis of GFP expression on tumor-infiltrating $CD8^+$ T cells at day 2 or day 4 post treatment with anti-PD-L1 or anti-PD-L1 and anti-CTLA-4. Non-treated and anti-PDL-1 (D2) (n=10), anti-PDL-1 (D4), anti-PDL-1/anti-CTLA-4 (D2), and anti-PDL-1/anti-CTLA-4 (D4) (n=9). **E-F**. Proportion of $CD8^+$ T cells expressing PD-1 following indicated therapies. Data points are shared for the 'control' and anti-PD-L1 groups between panels **E-F.** Data represented as the mean ± SEM. **E**. Non-treated n = 32, anti-PD-L1 n = 34, anti-CTLA-4 n = 11, P + C n = 12. **F**. n = 6 (non-treated) or 7 (carboplatin or anti-PD-L1) mice per group. **G**. Proportion of tumor-infiltrating $CD8^+$ T cells exhibiting a $PD\text{-}1^+A_{2A}R^-$ or $PD\text{-}1^+A_{2A}R^+$ phenotype with or without treatment with anti-PD-L1. n = 13 per group. *p<0.05, **p<0.01, ****p<0.0001, unpaired t test. Source data are provided as a Source Data file.

Supplementary Figure 4

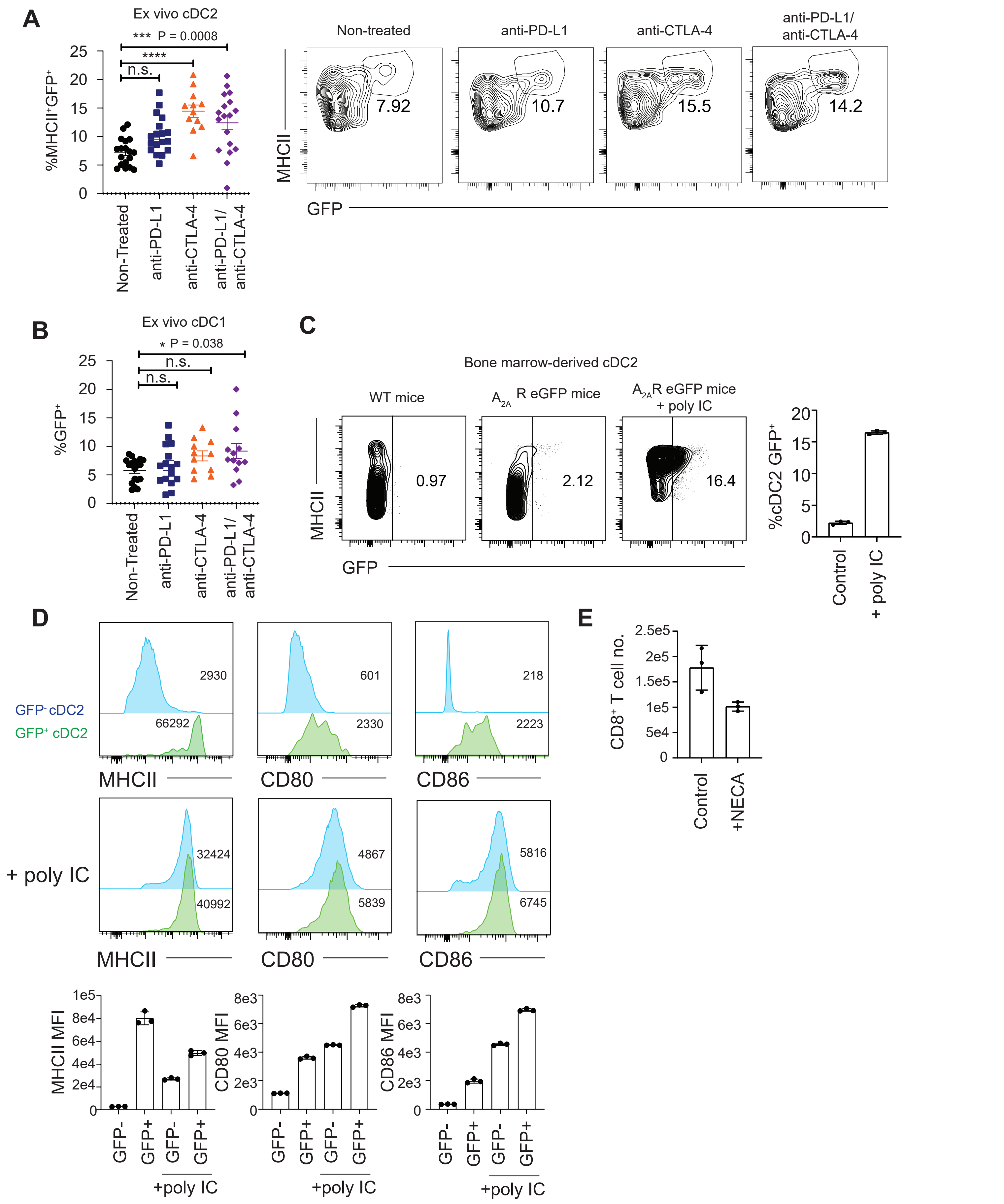

Supplementary Figure 4. Expression of $A_{2A}R$ is increased on activated cDC2s.

A-B. C57BL/6 $A_{2A}R$ GFP mice were injected with 5 x 10^5 AT3 ova tumor cells and treated where indicated with anti-PD-L1, anti-CTLA-4 or both anti-PD-L1 and anti-CTLA4 as per **Figure 4**. At day 7 post treatment the proportion of cDC2 (**A**) or cDC1 (**B**) expressing GFP was determined. Flow cytometry plots represent concatenated samples from one representative experiment. (**A**) Non-treated, anti-PD-L1, anti-PD-L1/anti-CTLA-4 (n=18) and anti-CTLA-4 (n=12). (**B**) Non-treated (n=18), anti-PD-L1 (n=16), anti-CTLA-4 (n=11) and anti-PD-L1/anti-CTLA-4 (n=13). Bar graphs represent mean ± SEM of 12-18 mice per group from 2-3 individual experiments.**C-E** Bone marrow derived dendritic cells were generated through culture of bone marrow cells in 150 ng/ ml Flt3L for 7 days. The expression of GFP, MHCII, CD80 and CD86 on MHCII⁺SIRPα⁺cDC2 cells was determined in the presence or absence of overnight stimulation with poly IC (25 µg/ ml). Flow cytometry plots and histograms represent concatenated samples from one representative experiment. Bar graphs represent mean ± SD from triplicate cultures. **E**. After generation of bone marrow derived DCs, cells were cultured overnight with poly IC and where indicated 1 µM NECA. DCs were then pulsed with 100nM SIINFEKL peptide for 4 hours prior and then 1e4 DCs were cocultured with 2e4 naïve OT-I T cells. $CD8^+$T cell numbers were then assessed at 72 hours post coculture. *$p<0.05$, ***$p<0.001$, ****$p<0.0001$, one way ANOVA (**A, B**). Source data are provided as a Source Data file.

Supplementary Figure 5

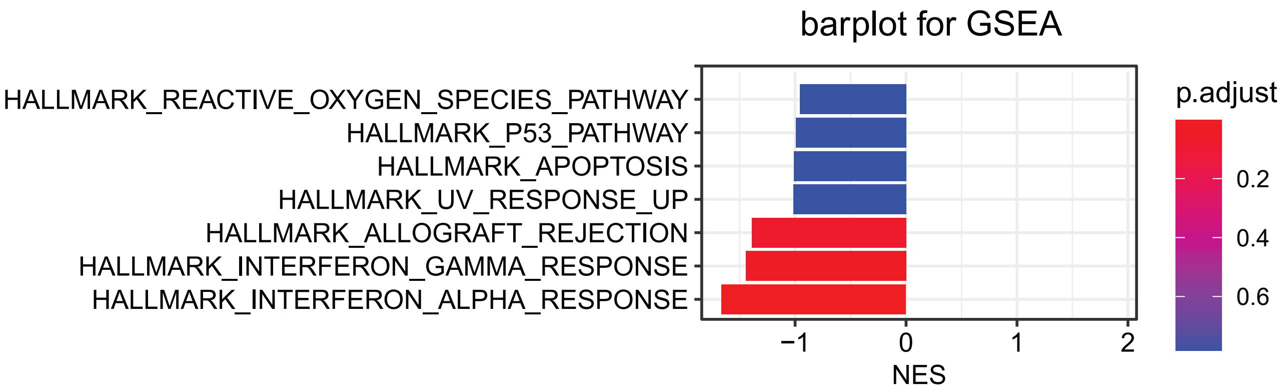

Supplementary Figure 5. Gene set enrichment analysis for $A_{2A}R^+$ $CD8^+$ T cells relative to $A_{2A}R^-$ $CD8^+$ T cells

RNA-Seq was performed on $CD8^+$ T cells isolated from AT3 ova tumors as per **Figure 5**. Unbiased pathway analysis for genes decreased in $A_{2A}R^+$ cells relative to $A_{2A}R^-$ cells. $CD8^+$ GFP^- (n = 3) and $CD8^+$ GFP^+ (n = 2) cells with biological samples pooled from n = 3 mice per replicate.

Supplementary Figure 6

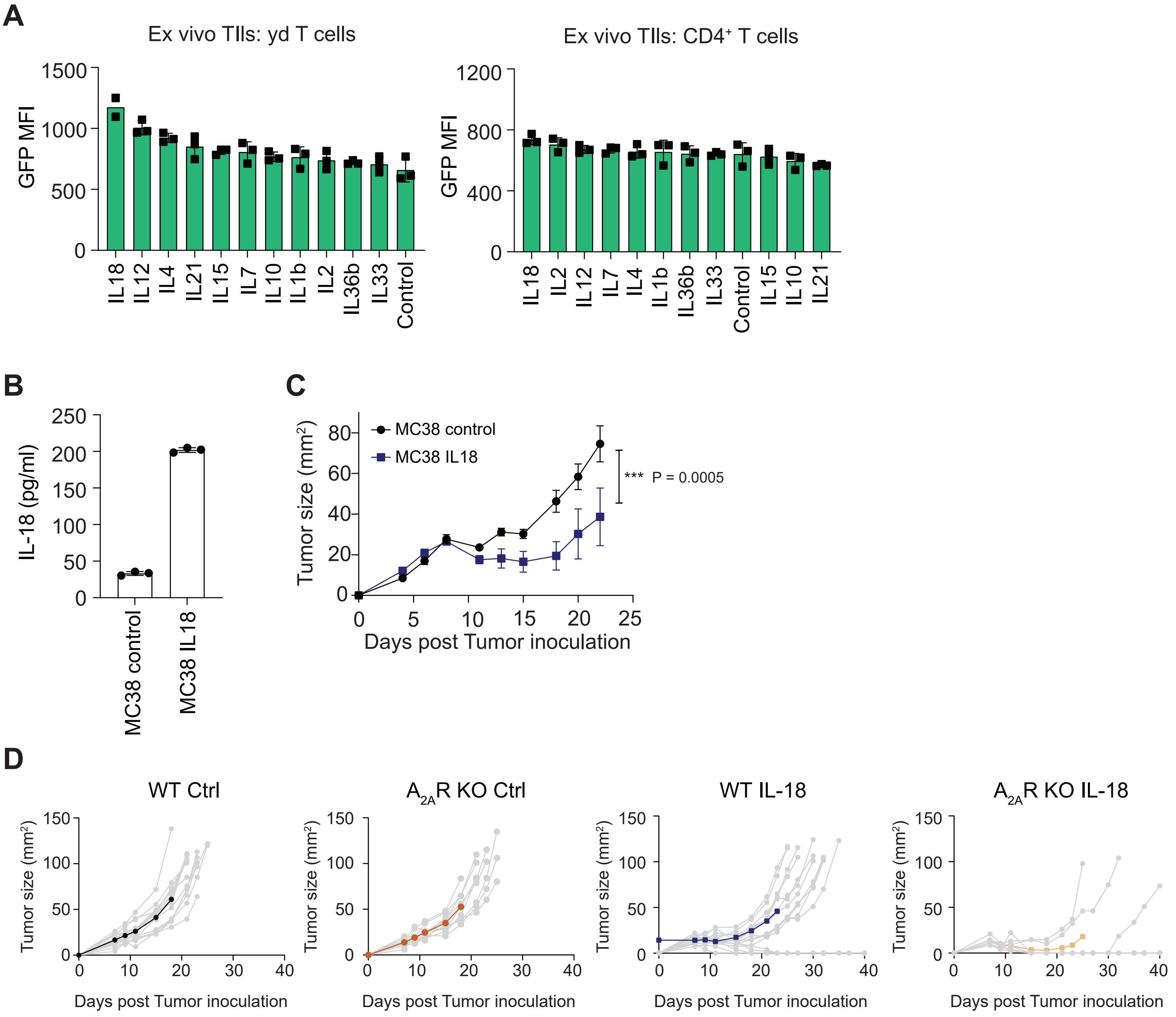

Supplementary Figure 6. Characterization of the impact of IL-18 on A_{2A}R expression and anti-tumor immunity

A. Tumor infiltrating lymphocytes were isolated from AT3 ova tumors and treated overnight with indicated cytokines (50 ng/ ml) as per **Figure 6**. Expression of GFP on indicated subsets was determined by flow cytometry. Data represents the mean ± SD of triplicate cultures. **B**. Supernatants derived from indicated MC38 tumor cells were assayed for IL-18 concentration by ELISA. Data represents the mean ± SD of 3 technical replicates. **C**. 1 x 10^6 MC38-mCherry or MC38-mCherry-IL-18 tumor cells were injected sub-cutaneously into C57BL/6 WT mice. Data is shown as the mean ± SEM of 7 (control) or 9 (IL-18) mice per group. ***p<0.001 2 way ANOVA.
D Individual tumor growth curves for mice from **Figure 6G-H**. Grey lines indicate individual mice, colored lines indicate the mean for each group. Source data are provided as a Source Data file.

**Flow Cytometry Antibodies**						
**Marker**	**Fluorochome**	**Dilution**	**Clone**	**Source**	**Catalogue number**	**Validation accessible via URL**
αGalCer tetramer	BV421	1:300	N/A	The Peter Doherty Institute for Infection and Immunity	N/A	N/A
Anti-Human/Mouse CD44	APC-R700	1:200	IM7	Thermo Fisher Scientific	56-0441-82	https://www.thermofisher.com/antibody/product/CD44-Antibody-clone-IM7-Monoclonal/56-0441-82
Anti-mouse CD103	BV711	1:100	2E7	BD Biosciences	748255	https://www.bdbiosciences.com/en-au/products/reagents/flow-cytometry-reagents/research-reagents/single-color-antibodies-ruo/bv711-hamster-anti-mouse-cd103.748255
Anti-mouse CD11c	BV785	1:150	N418	BioLegend	117336	https://www.biolegend.com/fr-fr/explore-new-products/brilliant-violet-785-anti-mouse-cd11c-antibody-7963?GroupID=BLG11937
Anti-mouse CD19	BV650	1:100	6D5	BioLegend	115541	https://www.biolegend.com/en-gb/products/brilliant-violet-650-anti-mouse-cd19-antibody-7851?GroupID=BLG10556
Anti-mouse CD19	PE	1:200	1D3	BD Biosciences	553786	https://www.bdbiosciences.com/en-au/products/reagents/flow-cytometry-reagents/research-reagents/single-color-antibodies-ruo/pe-rat-anti-mouse-cd19.553786
Anti-mouse CD25	APC	1:200	PC61.5	BioLegend	102012	https://www.biolegend.com/en-ie/products/apc-anti-mouse-cd25-antibody-420
Anti-mouse CD3	Alexa Fluor® 647	1:100	17A2	BD Biosciences	557869	https://www.bdbiosciences.com/en-au/products/reagents/flow-cytometry-reagents/research-reagents/single-color-antibodies-ruo/alexa-fluor-647-rat-anti-mouse-cd3-molecular-complex.557869
Anti-mouse CD39	PE-Cyanine7	1:150	24DMS1	Thermo Fisher Scientifc	25-0391-82	https://www.thermofisher.com/antibody/product/CD39-Antibody-clone-24DMS1-Monoclonal/25-0391-82
Anti-mouse CD4	BUV805	1:200	GK1.5	BD Biosciences	612900	https://www.bdbiosciences.com/en-au/products/reagents/flow-cytometry-reagents/research-reagents/single-color-antibodies-ruo/buv805-rat-anti-mouse-cd4.612900
Anti-mouse CD4	BV786	1:200	GK1.5	BD Biosciences	563727	https://www.bdbiosciences.com/en-au/products/reagents/flow-cytometry-reagents/research-reagents/single-color-antibodies-ruo/bv786-mouse-anti-human-cd4.563877
Anti-mouse CD45.2	APC-eF780	1:100	104	Thermo Fisher Scientific	47-0454-82	https://www.thermofisher.com/antibody/product/CD45-2-Antibody-clone-104-Monoclonal/47-0454-82
Anti-mouse CD62L	BUV737	1:200	MEL-14	BD Biosciences	612833	https://www.bdbiosciences.com/en-au/products/reagents/flow-cytometry-reagents/research-reagents/single-color-antibodies/buv737-rat-anti-mouse-cd62l.612833
Anti-mouse CD62L	BV786	1:100	MEL-14	BD Biosciences	564109	https://www.bdbiosciences.com/en-au/products/reagents/flow-cytometry-reagents/research-reagents/single-color-antibodies-ruo/bv786-rat-anti-mouse-cd62l.564109
Anti-mouse CD64	APC	1:100	X54-5/7.1	BioLegend	139306	https://www.biolegend.com/en-us/soluble-mhc/apc-anti-mouse-cd64-fcgammari-antibody-7874?GroupID=BLG8810
Anti-mouse CD69	Biotin	1:200	H1.2F3	BD Biosciences	553235	https://www.bdbiosciences.com/en-au/products/reagents/flow-cytometry-reagents/research-reagents/single-color-antibodies-ruo/biotin-hamster-anti-mouse-cd69.553235
Anti-mouse CD80	BUV496	1:150	16-10A1	BD Biosciences	741091	https://www.bdbiosciences.com/en-au/products/reagents/flow-cytometry-reagents/research-reagents/single-color-antibodies-ruo/buv496-hamster-anti-mouse-cd80.741091
Anti-mouse CD86	BV605	1:150	GL-1	BioLegend	105037	https://www.biolegend.com/en-us/products/brilliant-violet-605-anti-mouse-cd86-antibody-7798?GroupID=BLG11928
Anti-mouse CD8a	BV650	1:200	53-6.7	BioLegend	100742	https://www.biolegend.com/de-at/products/brilliant-violet-711-anti-mouse-cd8a-antibody-7926?GroupID=BLG279
Anti-mouse CD8a	BV711	1:200	53-6.7	BioLegend	100748 (500ul)	https://www.biolegend.com/en-gb/search-results/brilliant-violet-650-anti-mouse-cd8a-antibody-7635?GroupID=BLG2559
Anti-mouse F4/80 biotin	N/A	1:150	BM8	BioLegend	123106	https://www.biolegend.com/fr-ch/products/biotin-anti-mouse-f4-80-antibody-4066?GroupID=BLG5319
Anti-mouse IA/I-E (MHC II)	Alexa Fluor® 70	1:150	M5/114.15.2	BioLegend	107622	https://www.biolegend.com/en-us/cell-health/alexa-fluor-700-anti-mouse-i-a-i-e-antibody-3413
Anti-mouse LY108 (SLAMF6)	BV421	1:200	I3G3	BD Biosciences	740090	https://www.bdbiosciences.com/en-us/products/reagents/flow-cytometry-reagents/research-reagents/single-color-antibodies-ruo/bv421-mouse-anti-mouse-ly-108.740090
Anti-mouse Ly6C	BV605	1:200	HK1-4	BioLegend	128036	https://www.biolegend.com/en-gb/productstab/pe-cyanine7-anti-mouse-ly-6c-antibody-6063?Clone=HK1.4
Anti-mouse Ly6C	PE/Cyanine7	1:200	HK1-4	BioLegend	128018	https://www.biolegend.com/en-us/cell-health/brilliant-violet-605-anti-mouse-ly-6c-antibody-8727?GroupID=BLG7242
Anti-mouse Ly6G	PE	1:200	1A8	BD Biosciences	551461	https://www.bdbiosciences.com/en-eu/products/reagents/flow-cytometry-reagents/research-reagents/single-color-antibodies-ruo/pe-rat-anti-mouse-ly-6g.551461
Anti-mouse NK1.1	BV785	1:100	PK136	BioLegend	108749	https://www.biolegend.com/en-us/punchout/punchout-products/product-detail/brilliant-violet-785-anti-mouse-nk-1-1-antibody-10367?GroupID=GROUP20
Anti-mouse NK1.1	eFluor™ 450	1:100	PK136	Thermo Fisher Scientific	48-5941-82	https://www.thermofisher.com/antibody/product/NK1-1-Antibody-clone-PK136-Monoclonal/48-5941-82
Anti-mouse PD-1	BV711	1:150	29F.1A12	BioLegend	135231	https://www.biolegend.com/nl-be/cell-health/pe-anti-mouse-cd279-pd-1-antibody-6170?GroupID=BLG7930
Anti-mouse PD-1	PE	1:200	29F.1A12	BioLegend	135206	https://www.biolegend.com/de-at/search-results/brilliant-violet-711-anti-mouse-cd279-pd-1-antibody-12303
Anti-mouse TCRβ	APC	1:200	H57-597	BioLegend	109212	https://www.biolegend.com/en-gb/products/apc-anti-mouse-tcr-beta-chain-antibody-268?Clone=H57-597
Anti-mouse TCRβ	BUV737	1:100	H57-597	BD Biosciences	612821	https://www.bdbiosciences.com/en-au/products/reagents/flow-cytometry-reagents/research-reagents/single-color-antibodies/buv737-hamster-anti-mouse-tcr-chain.612821
Anti-mouse TCRβ	BV711	1:200	H57-597	BioLegend	109243	https://www.biolegend.com/en-us/search-results/brilliant-violet-711-anti-mouse-tcr-beta-chain-antibody-13539?GroupID=BLG6996
Anti-mouse TCRβ	eFluor™ 450	1:100	H57-597	Thermo Fisher Scientific	48-5961-82	https://www.thermofisher.com/antibody/product/TCR-beta-Antibody-clone-H57-597-Monoclonal/48-5961-82
Anti-mouse TCRγ/δ	PE/Cyanine7	1:100	GL3	BioLegend	118124	https://www.biolegend.com/de-de/clone-search/pe-cyanine7-anti-mouse-tcr-gamma-delta-antibody-7822?GroupID=BLG8973
Anti-mouse Thy1.2 (CD90.2)	PerCP	1:300	53-2.1	BioLegend	140316	https://www.biolegend.com/de-de/products/percp-anti-mouse-cd90-2-thy1-2-antibody-6807
Anti-mouse/rat XCR1	PerCP/Cyanine5.5	1:100	ZET	BioLegend	148208	https://www.biolegend.com/en-us/products/percp-cyanine5-5-anti-mouse-rat-xcr1-antibody-10397?GroupID=GROUP20
BUV395 Streptavidin	BUV395	1:1000	N/A	BD Biosciences	564176	https://www.bdbiosciences.com/en-au/products/reagents/flow-cytometry-reagents/research-reagents/single-color-antibodies-ruo/buv395-streptavidin.564176
iTAg^{TM} MHC Tetramer (H-2Kb OVA SIINFEKL-PE)	PE	1:50	N/A	MBL	TB-5001-1	https://www.mblintl.com/products/tb-5001-1/
MC38 tetramer (H-2Kb MuLV p15E Tetramer KSPWFTTL-PE)	PE	1:50	N/A	MBL	TB-M507-1	https://www.mblintl.com/products/tb-m507-1/
Rat anti-CD11b	BUV737	1:200	M1/70	BD Biosciences	612800	https://www.bdbiosciences.com/en-au/products/reagents/flow-cytometry-reagents/research-reagents/single-color-antibodies-ruo/buv737-rat-anti-cd11b.612800
Viability	Fixable yellow	1:400	N/A	Invitrogen	L34968A	https://www.thermofisher.com/order/catalog/product/L34968
**Immunofluorescence antibodies**						
**Marker**	**Fluorochome**	**Dilution**	**Clone**	**Source**	**Catalogue number**	**Validation accessible via URL**
Anti-GFP Polyclonal Antibody	Alexa Fluor™ 488	1:200	N/A	Thermo Fisher Scientific	A-21311	https://www.thermofisher.com/antibody/product/GFP-Antibody-Polyclonal/A-21311
Anti-mouse CD45.2	AF647	1:200	104	Biolegend	109818	https://www.biolegend.com/de-at/products/alexa-fluor-647-anti-mouse-cd45-2-antibody-3107?GroupID=BLG1934
Anti-mouse CD8a	AF594	1:200	53-6.7	Biolegend	100758	https://www.biolegend.com/de-at/products/alexa-fluor-594-anti-mouse-cd8a-antibody-9608?GroupID=BLG2559

**In vivo antibodies**						
			**Clone**	**Source**	**Catalogue number**	**Validation accessible via URL**
Anti asialo GM1 (Rabbit)				FUJIFILM Wako Pure Chemical Corporation	986-10001	https://labchem-wako.fujifilm.com/us/product/detail/W01W0198-1000.html
InVivoMAb anti-mouse CD8α			YTS 169.4	BioXcell	BE0117	https://bioxcell.com/invivomab-anti-mouse-cd8a-be0117
InVivoMAb anti-mouse CTLA-4 (CD152)			9H10	BioXcell	BE0131	https://bioxcell.com/invivomab-anti-mouse-ctla-4-cd152-be0131
InVivoMAb anti-mouse PD-L1 (B7-H1)			10F.9G2™	BioXcell	BE0101	https://bioxcell.com/invivomab-anti-mouse-pd-l1-b7-h1-be0101
InVivoMAb rat IgG2a isotype control			2A3	BioXcell	BE0089	https://bioxcell.com/invivomab-rat-igg2a-isotype-control-anti-trinitrophenol-be0089

**Other communal kits and reagents**						
		**Dilution**		**Source**	**Catalogue number**	**Validation accessible via URL**
BD Brilliant Stain Buffer Plus		1:100		BD Biosciences	566385	https://www.bdbiosciences.com/en-au/products/reagents/flow-cytometry-reagents/research-reagents/buffers-and-supporting-reagents-ruo/brilliant-stain-buffer-plus.566385

Supplementary Table 1. List of antibodies
