# Supplementary material for: A2AR eGFP reporter mouse enables elucidation of A2AR expression dynamics during anti-tumor immune responses
Source: Nat Commun. 2023 Nov 1;14:6990. doi: 10.1038/s41467-023-42734-0 (PMC10620403; doi:10.1038/s41467-023-42734-0)
Supplement: Supplementary file 3 — Reporting Summary [file 41467_2023_42734_MOESM3_ESM.pdf]

nature portfolio

Corresponding author(s): Paul A Beavis

Last updated by author(s): Oct 15, 2023

Reporting Summary

Nature Portfolio wishes to improve the reproducibility of the work that we publish. This form provides structure for consistency and transparency in reporting. For further information on Nature Portfolio policies, see our Editorial Policies and the Editorial Policy Checklist.

Statistics

For all statistical analyses, confirm that the following items are present in the figure legend, table legend, main text, or Methods section.

n/a	Confirmed	
☐	☒	The exact sample size (*n*) for each experimental group/condition, given as a discrete number and unit of measurement
☐	☒	A statement on whether measurements were taken from distinct samples or whether the same sample was measured repeatedly
☐	☒	The statistical test(s) used AND whether they are one- or two-sided<br>*Only common tests should be described solely by name; describe more complex techniques in the Methods section.*
☐	☒	A description of all covariates tested
☐	☒	A description of any assumptions or corrections, such as tests of normality and adjustment for multiple comparisons
☐	☒	A full description of the statistical parameters including central tendency (e.g. means) or other basic estimates (e.g. regression coefficient) AND variation (e.g. standard deviation) or associated estimates of uncertainty (e.g. confidence intervals)
☐	☒	For null hypothesis testing, the test statistic (e.g. *F*, *t*, *r*) with confidence intervals, effect sizes, degrees of freedom and *P* value noted<br>*Give P values as exact values whenever suitable.*
☒	☐	For Bayesian analysis, information on the choice of priors and Markov chain Monte Carlo settings
☒	☐	For hierarchical and complex designs, identification of the appropriate level for tests and full reporting of outcomes
☒	☐	Estimates of effect sizes (e.g. Cohen's *d*, Pearson's *r*), indicating how they were calculated

Our web collection on statistics for biologists contains articles on many of the points above.

Software and code

Policy information about availability of computer code

Data collection	Flow cytometry: BD FacsDiva version 8 (FlowJo LLC)<br>RNA-seq: CASAVA v1.8.2, Cutadapt v2.1, FastQC v0.11.6, RNA-SeQC v1.1.8
Data analysis	Flow cytometry: FlowJo version 10 (FlowJo LLC)<br>Differentially expressed gene analysis: EdgeR (https://bioconductor.org/packages/release/bioc/html/edgeR.html)<br>Gene set enrichment analysis: Enrichr (http://amp.pharm.mssm.edu/Enrichr)<br>General: Microsoft Excel 2010<br>Statistical analysis and data presentation: Graphpad Prism 9<br>Gene expression analysis: featureCounts, Rsubread 2.10.5; heatmaps: pheatmap R package

For manuscripts utilizing custom algorithms or software that are central to the research but not yet described in published literature, software must be made available to editors and reviewers. We strongly encourage code deposition in a community repository (e.g. GitHub). See the Nature Portfolio guidelines for submitting code & software for further information.

Data

Policy information about availability of data

All manuscripts must include a data availability statement. This statement should provide the following information, where applicable:

- Accession codes, unique identifiers, or web links for publicly available datasets
- A description of any restrictions on data availability
- For clinical datasets or third party data, please ensure that the statement adheres to our policy

All raw data is contained within the Source Data file. Source data are provided with this paper. The RNA-sequencing data used in the study have been deposited in the Gene Expression Omnibus under the accession code GSE230135 available at https://www.ncbi.nlm.nih.gov/geo/query/acc.cgi?acc=GSE230135.

Research involving human participants, their data, or biological material

Policy information about studies with human participants or human data. See also policy information about sex, gender (identity/presentation), and sexual orientation and race, ethnicity and racism.

Reporting on sex and gender	Sex and gender were not considered in the study design for in vitro experiments using human PBMCs obtained from the Red Cross. This information was not collected.
Reporting on race, ethnicity, or other socially relevant groupings	Race and ethnicity were considered in the study design for in vitro experiments using human PBMCs obtained from the Red Cross. This information was not collected.
Population characteristics	The covariate population characteristics for in vitro studies with human PBMCs was not collected.
Recruitment	Healthy donor PBMCs were obtained from buffy packs obtained from the Australian Red Cross.
Ethics oversight	Ethics for the study was approved by the Peter MacCallum Cancer Centre Human Research Ethics committee.

Note that full information on the approval of the study protocol must also be provided in the manuscript.

Field-specific reporting

Please select the one below that is the best fit for your research. If you are not sure, read the appropriate sections before making your selection.

☒ Life sciences ☐ Behavioural & social sciences ☐ Ecological, evolutionary & environmental sciences

For a reference copy of the document with all sections, see nature.com/documents/nr-reporting-summary-flat.pdf

Life sciences study design

All studies must disclose on these points even when the disclosure is negative.

Sample size	Experiments were performed with sufficient power to achieve statistical significance based upon an effect size of 30%, which would have been deemed clinically significant. All therapeutic experiments were performed with a minimum of 6 mice per group.
Data exclusions	No data were excluded from the manuscript.
Replication	All experiments were replicated in at least 2 independent experiments.
Randomization	Mice were randomized prior to treatment according to tumor size to ensure all groups had equivalent tumor burden prior to therapy. Groups were age and sex matched. Randomization was not relevant to in vitro studies because experiments involved one population of cells divided into different treatment groups.
Blinding	Groups we re not blinded because the same investigators who performed the experiments were responsible for randomizing the groups.

Reporting for specific materials, systems and methods

We require information from authors about some types of materials, experimental systems and methods used in many studies. Here, indicate whether each material, system or method listed is relevant to your study. If you are not sure if a list item applies to your research, read the appropriate section before selecting a response.

Materials & experimental systems

n/a	Involved in the study	
	x	Antibodies
	x	Eukaryotic cell lines
x		Palaeontology and archaeology
	x	Animals and other organisms
x		Clinical data
x		Dual use research of concern
x		Plants

Methods

n/a	Involved in the study	
x		ChIP-seq
	x	Flow cytometry
x		MRI-based neuroimaging

Antibodies

Antibodies used

Antibodies used in this study were obtained from commercial suppliers (BD Biosciences, Biolegend, Thermo Scientific, BioXcell, MBL or FUJIFILM Wako Pure Chemical Corporation). A list of relevant information on the antibodies (Supplier, Catalog number, clone, dilution and product URL) is provided in a Supplementary Table.

αGalCer tetramer, The Peter Doherty Institute for Infection and Immunity 1:300
BUV395 Streptavidin, BD Biosciences 564176 1:1000
Anti-mouse CD103, Clone 2E7, BD Biosciences 748255 1:100
Rat anti-CD11b, Clone M1/70, BD Biosciences 612800 1:200
Anti-mouse CD11c, Clone N418, BioLegend 117336 1:150
Anti-mouse CD19, Clone 6D5, BioLegend 115541 1:100
Anti-mouse CD19, Clone 1D3, BD Biosciences 553786 1:200
Anti-mouse CD25, Clone PC61.5, BioLegend 102012 1:200
Anti-mouse CD3, Clone 17A2, BD Biosciences 557869 1:100
Anti-mouse CD39, Clone 24DMS1, Thermo Fisher Scientific 25-0391-82 1:150
Anti-mouse CD4, Clone GK1.5, BD Biosciences 612900 1:200
Anti-mouse CD4, Clone GK1.5, BD Biosciences 563727 1:200
Anti-Human/Mouse CD44, Clone IM7, Thermo Fisher Scientific 56-0441-82 1:200
Anti-mouse CD45.2, Clone 104, Thermo Fisher Scientific 47-0454-82 1:100
Anti-mouse CD62L, Clone MEL-14, BD Biosciences 612833 1:200
Anti-mouse CD62L, Clone MEL-14, BD Biosciences 564109 1:100
Anti-mouse CD64, Clone X54-5/7.1, BioLegend 139306 1:100
Anti-mouse CD69, Clone H1.2F3, BD Biosciences 553235 1:200
Anti-mouse CD80, Clone 16-10A1, BD Biosciences 741091 1:150
Anti-mouse CD86, Clone GL-1, BioLegend 105037 1:150
Anti-mouse CD8a, Clone 53-6.7, BioLegend 100742 1:200
Anti-mouse CD8a, Clone 53-6.7, BioLegend 100748 (500ul) 1:200
Anti-mouse F4/80 biotin, Clone BM8, BioLegend 123106 1:150
Anti-mouse IA/I-E (MHC II), Clone M5/114.15.2, BioLegend 107622 1:150
iTAgTM MHC Tetramer (H-2Kb OVA SIINFEKL-PE), MBL, TB-5001-1 1:50
Anti-mouse LY108 (SLAMF6), Clone I3G3, BD Biosciences 740090 1:200
Anti-mouse Ly6C, Clone HK1-4, BioLegend 128036 1:200
Anti-mouse Ly6C, Clone HK1-4, BioLegend 128018 1:200
Anti-mouse Ly6C, Clone 1A8, BD Biosciences 551461 1:200
MC38 tetramer (H-2Kb MuLV p15E Tetramer KSPWFTTL-PE), MBL TB-M507-1 1:50
Anti-mouse NK1.1, Clone PK136, BioLegend 108749 1:100
Anti-mouse NK1.1, Clone PK136, Thermo Fisher Scientific 48-5941-82 1:100
Anti-mouse PD-1, Clone 29F.1A12, BioLegend 135231 1:150
Anti-mouse PD-1, Clone 29F.1A12, BioLegend 135206 1:200
Anti-mouse TCRβ, Clone H57-597, BioLegend 109212 1:200
Anti-mouse TCRβ, Clone H57-597, BD Biosciences 612821 1:100
Anti-mouse TCRβ, Clone H57-597, BioLegend 109243 1:200
Anti-mouse TCRβ, Clone H57-597, Thermo Fisher Scientific 48-5961-82 1:100
Anti-mouse TCRγ/δ, Clone GL3, BioLegend 118124 1:100
Anti-mouse Thy1.2 (CD90.2), Clone 53-2.1, BioLegend 140316 1:300
Viability, Invitrogen L34968A 1:400
Anti-mouse/rat XCR1, Clone ZET, BioLegend 148208 1:100
Anti-mouse CD45.2, Clone 104, Biolegend 109818 1:200
Anti-mouse CD8a, Clone 53-6.7, Biolegend 100758 1:200
Anti-GFP Polyclonal Antibody, Thermo Fisher Scientific A-21311 1:200
InVivoMAb rat IgG2a isotype control, Clone 2A3, BioXcell BE0089
InVivoMAb anti-mouse PD-L1 (B7-H1), Clone 10F.9G2™, BioXcell BE0101
InVivoMAb anti-mouse CTLA-4 (CD152), Clone 9H10, BioXcell BE0131
InVivoMAb anti-mouse CD8α, Clone YTS 169.4, BioXcell BE0117

	Anti asialo GM1 (Rabbit), FUJIFILM Wako Pure Chemical Corporation 986-10001<br>BD Brilliant Stain Buffer Plus, BD Biosciences 566385 1:100
Validation	All antibodies were validated by the supplier. Relevant information can be found in the Supplementary Table

Eukaryotic cell lines

Policy information about cell lines and Sex and Gender in Research

Cell line source(s)	AT3 tumor cells were obtained from Dr. Trina Stewart and engineered to express chicken ovalbumin. MC38 tumor cells were obtained from Dr. Nicole Haynes
Authentication	Cell lines were not authenticated but were uitiized within 10 passages of a master stock
Mycoplasma contamination	All lines were tested negative for mycoplasma contamination
Commonly misidentified lines<br>(See ICLAC register)	None of the cell lines are listed on the ICLAC database

Animals and other research organisms

Policy information about studies involving animals; ARRIVE guidelines recommended for reporting animal research, and Sex and Gender in Research

Laboratory animals	C57BL/6 mice and transgenic mice were utilized where indicated. Mice were used between 6-16 weeks of age.
Wild animals	This study did not involve wild animals.
Reporting on sex	Studies utilizing AT3ova were performed in female mice. Studies using MC38 were performed in male or female mice.
Field-collected samples	N/A.
Ethics oversight	Ethics oversight was performed by the Peter MacCallum Cancer Centre Animal Experimentation Ethics Committee (AEEC).

Note that full information on the approval of the study protocol must also be provided in the manuscript.

Flow Cytometry

Plots

Confirm that:

☐ The axis labels state the marker and fluorochrome used (e.g. CD4-FITC).

☒ The axis scales are clearly visible. Include numbers along axes only for bottom left plot of group (a 'group' is an analysis of identical markers).

☒ All plots are contour plots with outliers or pseudocolor plots.

☒ A numerical value for number of cells or percentage (with statistics) is provided.

Methodology

Sample preparation	Spleen samples were treated twice or once respectively with ACK lysis buffer before staining for flow cytometry. Tumors were digested in SAFC DMEM media with 0.01 mg/mL DNase (Sigma Aldrich) and 1 mg/mL type IV collagenase for 30 minutes at 37oC. Following digestion, tumor samples were filtered twice through a 70 μm filter to create a single cell suspension and resuspended in Fc block prior to staining for analysis by flow cytometry.
Instrument	FACS data were obtained on a BD FACS Symphony A5 and BD LSRFortessa X-20 from the Peter MacCallum Cancer Centre flow cytometry core facility.
Software	Data was analyzed using Flowjo software 10.8.1
Cell population abundance	For experiments where cells with FACS sorted, re-analysis was performed to confirm sort purity
Gating strategy	FSC/SSC gate was first used to gate on the morphology of leukocytes. A singlet gates was next used (FSC-A vs FSC-h) to exclude doublets followed by a viability gate (Fixable Yellow) to excluded dead cells.

☒ Tick this box to confirm that a figure exemplifying the gating strategy is provided in the Supplementary Information.
